# How Confident Are We at Assessing and Managing Fatigue in Palliative Care Patients? A Multicenter Survey Exploring the Current Attitudes of Palliative Care Professionals

**DOI:** 10.1089/pmr.2020.0005

**Published:** 2020-05-28

**Authors:** Gemma Ingham, Katalin Urban

**Affiliations:** Department of Palliative Medicine, Prince of Wales Hospital, Randwick, New South Wales, Australia.

**Keywords:** attitudes, fatigue, exercise, palliative care, palliative medicine, quality of life, surveys

## Abstract

***Background:*** Fatigue is a common and significant problem for palliative care (PC) patients, affecting up to 80% of patients. Health care professionals (HCPs) commonly underestimate its significance and lack the confidence in how to manage it, resulting in poor quality of life. It is currently not known how PC professionals manage fatigue in clinical practice or what the barriers to implementation are.

***Objective:*** To determine the current attitudes of HCPs toward fatigue management in patients with a life-limiting illness.

***Design/Setting:*** An electronic survey, created on REDCap, was distributed to all staff working directly with PC patients in both the community and inpatient setting within the Sydney Local Health District. The study duration was for four weeks (May 1–30, 2018).

***Results:*** Participants recognized that fatigue is common, but only 58.5% recognized that severe fatigue is more distressing than pain. A total of 77.2% of participants do not find fatigue an easy symptom to manage and less than half (46.9%) feel confident assessing and managing it. There was no consistent systematic approach to management although exercise, education, and pacing/energy conservation were recognized as important interventions. Themes identified as potential barriers to management included lack of resources, poor patient and staff understanding, and patient/disease factors.

***Conclusions:*** HCPs lack confidence in assessing fatigue and completing an individualized management plan; the approaches adopted are highly variable. This justifies the need for further education, as well as further research assessing the efficacy of a multimodal intervention and a guideline to assist in management.

## Introduction

Fatigue is one of the most common symptoms encountered by palliative care (PC) patients, affecting >80% of patients,^1^ and the most associated with moderate/severe distress.^2^ It is defined as “the subjective feeling of tiredness, weakness or lack of energy”^1^ and can affect people physically, emotionally, and cognitively, and those with cancer and noncancer diagnoses. It is frequently underrecognized, significantly impacting on a patient's quality of life, independence, and dignity.

Both patients and health care professionals (HCPs) perceive fatigue as a normal feature of an advanced, life-limiting illness and that it has to be endured. Patient surveys confirm that it is underreported, and as treatment options are perceived as scarce, management advice remains scant and variable. Stone et al. completed a questionnaire-based study of cancer patients attending the outpatient department or day unit in the United Kingdom and found that fatigue affected 58% of the 576 participants, significantly affecting their day-to-day life more than any other symptom. Effects included impact on their ability to work, to enjoy life, to take care of their family, and their emotional well-being. Less than half had reported it to their HCP, most commonly as they thought it was inevitable, and only 14% had been recommended any treatment.^3^ Although HCPs recognize fatigue as a problem, many do not realize its significance. A U.S. study by Vogelzang et al. found that 61% of patients reported fatigue affecting their daily life more than pain, whereas only 37% of oncologists believed this was true; the vast majority (61%) thought pain was a bigger problem.^4^

Fatigue remains a very challenging symptom to manage effectively as a result of overlapping disease-specific symptoms and the lack of evidence for which intervention is most efficacious for fatigue in the PC setting.^1^ There is currently no definitive international guideline, but current literature suggests that a multimodal multidisciplinary intervention holds the greatest potential for effective management. This includes education, individual exercise program, cognitive-behavioral therapy (CBT), and energy conservation and restoration strategies, such as sleep and nutrition counseling.^5^ There is no evidence to support pharmacological interventions.^6^ Despite this, the Palliative Care Outcomes Collaborative (PCOC) reports that in Australia, community PC services are failing to meet the benchmarks set for this symptom.^2^ For example, between January and June 2018 within the Sydney Local Health District (SLHD), only 20.9% of patients had a reduction in their distress scores from moderate/severe at the start of the phase to absent/mild at the phase end, significantly <60% benchmark set by PCOC.^7^

The European Association of Palliative Care's (EAPC) consensus group has recognized that HCPs have “inadequate assessment skills and insufficient knowledge about multidimensional treatment options” of fatigue.^1^ Pearson et al. distributed an electronic survey to 129 HCPs within the oncology setting. Over half of respondents screened for fatigue, but only a quarter went on to conduct in-depth cancer-related fatigue (CRF) assessments. Less than one-quarter used a clinical guideline, and awareness of interventions was poor with only a quarter able to list five appropriate interventions. There was a perception that CRF was inadequately managed and that there is a need for improved guidelines, enhanced expertise, and better access to services.^8^

Despite the significant incidence of fatigue in the PC setting and the detrimental effect it has on patients' quality of life, it is not clear how HCPs currently manage fatigue in this specific setting, nor whether multimodal interventions can feasibly be integrated into patient care. To the best of our knowledge, this is the only survey in the literature that explores PC HCP perceptions of fatigue, including its importance, their current assessment and treatment strategies, and some of the difficulties they may have encountered. The primary research aim is to better understand the current attitudes and confidence of PC HCPs toward fatigue, whether their management is consistent and evidence based, and what barriers need to be overcome to improve future management. Future education, guidelines, research, and resource allocation can therefore be appropriately targeted to improve patient outcomes.

## Method

### Data collection

Participant selection was purposive using typical case sampling technique.^9^ Study participants were identified by a clinical lead at each research site and included all medical, nursing, and allied health PC staff within the SLHD. This included five community health care centers, one palliative care unit (PCU), and two PC ward teams. Other HCPs involved in the care of PC patients but working outside of these specific settings were not included (i.e., those whose primary role is not delivering specialist PC).

Study duration was for four weeks from May 1 to 30, 2018. An e-mail inviting staff to participate was sent on day one and a reminder was sent weekly for the study duration. All participants were voluntary and remained anonymous throughout. Implied consent was assumed by completing the survey.

The survey was created on REDCap (“Research Electronic Data Capture,” a secure web-based data capture and data management software tool). Information about the survey was provided before starting. Questions were created based on current literature and included basic demographic information and questions on current fatigue management. Before distribution, feedback on the survey design was received from a small committee of HCPs (medical, nursing, and allied health representatives).

### Statistical analysis

REDCap was used to both store the deidentified data and interpret them using the REDCap data report generation tool. These basic descriptive statistics were collated in an excel document. Incomplete surveys were excluded from analysis. Those questions with free-text answers were reviewed using a grounded theory by two lead investigators. Themes/categories were initially identified and indexed for each answer. Investigators then systematically compared answers to establish analytical categories that were later refined and grouped together.^10^

### Ethics approval

A Human Research Ethics Application (HREA) was approved by the SLHD Ethics Review Committee (RPAH Zone) (HREC reference LNR/17/PRAH/662) on January 30, 2018, as were four site-specific applications (SSAs).

## Results

### Demographic information

One hundred sixty-four surveys were distributed in total, with 66 (40%) returned fully completed. Participants were predominantly female (87.9%) and employed as a nurse (77.3%) (including both specialist and generalist/community PC nurses). All work settings were represented, with the greatest proportion having community exposure (community *n* = 42, PCU *n* = 21, and medical ward *n* = 13). Most participants had at least two years of PC experience (86.3%). Table 1 summarizes their demographic information; this is representative of the expected demographics of PC HCPs within the SLHD.

**Table 1.** Demographic Data of Participants

	*n* = 66 (%)
Sex	
Male	8 (12.1)
Female	58 (87.9)
Clinical role	
Palliative medicine specialist	7 (10.6)
Palliative medicine trainee	3 (4.5)
Junior doctor	0
Nurse	51 (77.3)
Allied health worker	5 (7.6)
Other	0
Place of work	
Community only	38 (57.6)
PCU only	14 (21.2)
Medical ward only	5 (7.6)
PCU and medical ward	5 (7.6)
Community and medical ward	2 (3.0)
Community and PCU	1 (1.5)
All settings	1 (1.5)
Length of experience (years)	
<1	9 (13.6)
2–5	28 (42.4)
>5	29 (43.9)

PCU, palliative care unit.

### Attitudes toward fatigue

The majority of participants recognized that fatigue is common and part of the natural history of a chronic life-limiting illness (86.4% agree or strongly agree), but only 58.5% recognized that patients find severe fatigue more distressing than pain (Table 2). A total of 77.2% of participants do not find fatigue an easy symptom to manage and less than half (46.9%) feel confident assessing and managing it. Responses were consistent across all participant groups; doctors, nurses, and allied health staff similarly do not find it an easy symptom to manage, nor feel confident in its assessment and management.

**Table 2.** Participants' Attitudes toward Fatigue

	“Fatigue is common in palliative care patients,” *n* = 66 (%)	“Fatigue is an inevitable part of a chronic life-limiting illness,” *n* = 66 (%)	“Palliative care patients consider severe fatigue more distressing than pain,” *n* = 65 (%)	“I feel confident assessing and managing fatigue,” *n* = 66 (%)	“Fatigue is an easy symptom to manage,” *n* = 66 (%)
Strongly agree	51 (77.3)	18 (27.3)	10 (15.4)	8 (12.1)	0
Agree	15 (22.7)	39 (59.1)	28 (43.1)	23 (34.8)	5 (7.6)
Undecided	0	7 (10.6)	16 (24.6)	26 (39.4)	10 (15.2)
Disagree	0	2 (3)	10 (15.4)	9 (13.6)	36 (54.5)
Strongly disagree	0	0	1 (1.5)	0	15 (22.7)

### Approach to management

There was no consensus on the amount of time participants felt they needed or could allocate to perform a full fatigue assessment and then initiate a management plan (60% reported requiring <15 minutes, with the remainder requiring longer). A total of 64.4% reported having the exact or more time than required. Only 7.7% reported having no time due to other priorities.

The 54 responses to the question “what is your general approach to managing fatigue?” were hugely variable (12 participants chose not to answer); this was particularly true of the responses by nursing staff, with the majority showing no consistent systematic approach. An example of a more detailed response is as follows:

*1. Assess regularly using symptom assessment scale (SAS) and brief fatigue inventory (BFI).*
*2. Pay attention to the patient's experience of fatigue— listening carefully and validating their distress can reduce the distress associated with fatigue.*
*3. For patients with a good prognosis—reverse all reversible causes, exercise as appropriate, sleep hygiene, daytime stimulation, assess and manage other symptoms, provide emotional support/counselling/stress management, equipment/occupational therapy (OT) review, treat depression, judicial use of dexamethasone e.g., short-term use to allow a patient to attend a wedding* etc., *trial of psychostimulant.*
*4. For patients who are approaching end stage—energy conservation.*

This compares with the more frequent, limited responses of:

*Other than energy conserving techniques I do not know how else to manage fatigue*

*Taking more short rest breaks throughout the day.*

All approaches, although not evidence based, were reasonable (none was considered unsafe) and when combined can be summarized as follows:

1. Fatigue assessment including when, severity, level of distress, and impact on patient's function.
2. Assess for reversible/contributing factors and treat as appropriate, for example, medications, depression, other symptoms.
3. Address patients' understanding of disease (including its impact on fatigue) and their expectations (including goal setting).
4. Make individualized management plan.
5. Encourage regular exercise.
6. Energy conservation and pacing.
7. Allied health referral—OT for energy conservation, pacing techniques, equipment; physiotherapist (PT) for individualized exercise program; psychologist for psychological support including CBT.
8. Regular follow-up and review of outcomes.

There was a general consensus about the importance of exercise and pacing/energy conservation, and of addressing patient's expectations, but otherwise no clear themes were identified. Although outside the scope of the question, only one participant gave specific examples of exercises he or she would suggest. There was no agreement by the doctors about the benefits of medication adjuncts, such as dexamethasone and methamphetamine; four reported that they would consider their use, two stated they would rarely use it, and the remaining four did not comment.

### Barriers to management

Table 3 illustrates the eight themes that were identified as potential difficulties to successful management of fatigue. They were consistently mentioned by all staff working in all sectors. Fourteen participants chose not to answer.

**Table 3.** Themes Identified As Potential Difficulties to Successful Management of Fatigue

Themes	Quotes
1. Lack of resources and time restraints	*Time limitations—priority is often to discharge patients rather than considering and giving time to developing a fatigue management plan*
	*Needs an interdisciplinary management plan which is not always available*
	*Not enough resources especially allied health*
	*Sometimes appropriate equipment not available when needed*
	*Access to OTs to discuss energy conservation techniques*
	*Could be discussed more in reviews at group meetings*
	*Difficult to implement CBT unless psychologist available*
2. Poor compliance and motivation	*The client may not follow through with plan if no immediate gain*
	*Clients not wanting to try different solutions to improve their fatigue. People don't like change in behaviour and also want instant change, will not work at obtaining change.*
	*Patients feel psychologically ‘resigned’ or have ‘given up’*
	*If they have been experiencing it for a while, they can be less willing to try alternate methods of doing things*
	*Fatigue itself makes it difficult to motivate the person*
3. Patient/disease factors	*Ability of the person to try things which may help (exercise* etc.*)*
	*The extent/or degree of fatigue has an impact e.g., if they can't get out of bed due to fatigue it can be more difficult than someone who can no longer walk 2 km*
	*Barriers including CALD, delirium, dementia*
	*Patient feeling too unwell*
	*Short/limited windows of time to address fatigue if patients need to rest for long periods throughout the day*
4. Expectation that it is unavoidable	*For many patients as the disease continues to progress fatigue can become unavoidable.*
	*No real ‘cure’ as such, just ways of managing*
	*Patient or family expectations that it is inevitable they have fatigue.*
5. Significant distress	*It is often the most difficult to manage patients distress over this.*
	*Psychological issues—distress with cancer diagnosis and pain being barriers*
	*Dealing with the distress fatigue can bring on in patients and their families*
	*Patients often blame themselves as being ‘lazy’ or ‘not positive enough’*
	*Patients feeling loss of self and meaning due to fatigue*
6. Patients' understanding of what fatigue is, including use of assessment tools	*There is difficulty for patients in seeing the difference between the severity of the fatigue and the distress caused by the fatigue, leading them to score fatigue high on the SAS which does not reflect any changes to the distress resulting from interventions*
	*Getting the patient to understand what fatigue is*
7. Limited management options and uncertainty about what to recommend	*Unsure what exercises to prescribe*
	*My own knowledge deficit about the symptom*
	*No good pharmacotherapy available*
	*The management strategies often don't remove fatigue and only offer some relief if any*
	*There is not much information to manage fatigue*
	*Patients are seen by less experienced staff who don't know how to manage or acknowledge fatigue*
	*Not much (apart from exercise in those who are still well enough to do it) is effective*
	*Hard to refer to someone professional on fatigue*
	*Ad hoc recommendations between health professionals, not a lot of literature or patient education material*
	*Need to use some medications for other symptoms that may exacerbate fatigue. Often more refractory to strategies than other symptoms*
8. Coexisting symptoms	*Differentiating pain and fatigue*
	*Identifying it as a stand-alone symptom can be problematic (fatigue vs. drowsiness from other medications like opioids vs. psychological/psychiatric issues related to end of life)*
	*Other factors involved affecting the management of fatigue, such as symptoms, that patients view as more important to be addressed than fatigue*
	*Need to use some medications for other symptoms that may exacerbate fatigue*

CALD, culturally and linguistically diverse; CBT, cognitive-behavioral therapy; OT, occupational therapy; SAS, symptom assessment scale.

### Specific strategies

**FIG. 1.**
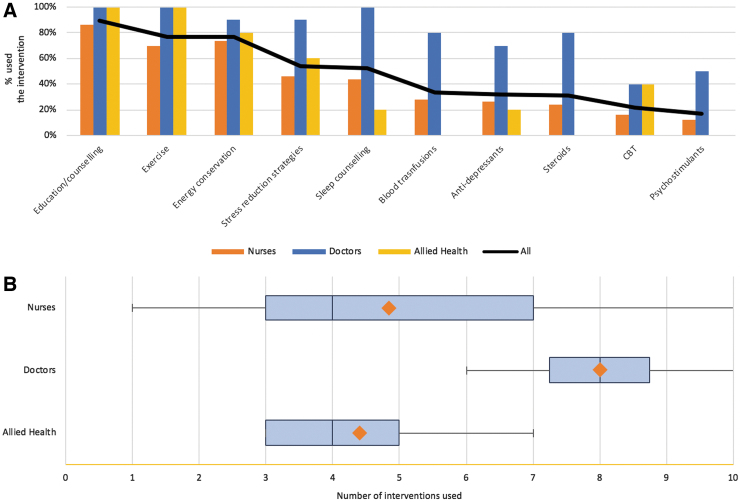
**(A)** Chart showing the percentage of participants reported to have previously used each specific intervention. **(B)** Box plot showing the number of interventions (range, first/third quartile, median, and mean) used by each professional group.

Most participants recognized the importance of exercise, OT referral, and psychology review, although most only agreed with the statement as opposed to strongly agreed (Table 4). There was uncertainty about whether patients should be advised to rest more. Figure 1 demonstrates the different strategies used. Education (89.2%), exercise (76.9%), and energy conservation (76.9%) were the most commonly used strategies, whereas steroids (30.8%), CBT (21.5%), and psychostimulants (16.9%) were used infrequently. Doctors use a greater number of interventions than nursing or allied health staff.

**Table 4.** Participants' Opinions on Specific Fatigue Management Strategies

	“Patients who feel fatigued should be advised to rest more,” *n* = 66 (%)	“Exercise is a treatment for fatigue,” *n* = 66 (%)	“Referral for OT has a role in fatigue management,” *n* = 66 (%)	“Patients with fatigue may benefit from a psychology review,” *n* = 64 (%)
Strongly agree	1 (1.5)	15 (22.7)	22 (33.3)	17 (26.6)
Agree	24 (36.4)	42 (63.6)	35 (53.0)	43 (67.2)
Undecided	15 (22.7)	5 (7.6)	8 (12.1)	3 (4.7)
Disagree	24 (36.4)	4 (6.1)	1 (1.5)	1 (1.6)
Strongly disagree	2 (3.0)	0	0	0

OT, occupational therapy.

## Discussion

We believe that the cohort of participants in this survey-based study is representative of the typical level of PC support in many metropolitan networks in Australia. Despite recognizing the frequency of fatigue in life-limiting illnesses, our results support current literature; HCPs believe it is an inevitable part of a life-limiting illness but do not recognize the level of severe distress it causes PC patients. This correlates to what is described in studies by Vogelzang et al. and Williams et al., both of which describe that HCPs underestimate the impact of CRF, perceiving that pain has greater impact on their daily lives.^4,11^ It also supports the assumption that HCPs lack the confidence in how to manage it adequately. This was seen universally in all professional groups in our study; doctors, nurses, and allied health staff all agree that it is not an easy symptom to manage, nor feel confident in its assessment and management. It is therefore not surprising that as reported in Stone et al.'s study, patients report being infrequently screened for fatigue, nor receive advice on how to manage it, but that they also infrequently report it to their HCPs.^3^ Passik et al. report that the most common reason for poor patient communication about fatigue is the HCPs' failure to offer interventions (47%) and the patients' lack of awareness of effective treatments for fatigue (43%).^12^ This correlates to many of the reported barriers in this study.

All of the reported approaches to management were sensible, but there was no consensus approach, with huge variability in adopted strategies, not all of which were evidence based. Although the summary of combined approaches is similar to the National Comprehensive Cancer Network (NCCN) guidelines,^13^ this algorithm was only achievable when combining the knowledge of multiple individuals, emphasizing the need for further education and local guidelines. Similarly, Pearson et al. reported that in a survey of HCPs who work with cancer patients (medical, nursing, and allied health professionals), less than one-quarter use a clinical guideline, and that awareness of interventions was variable with only a quarter able to list five appropriate interventions for CRF.^8^

The barriers described also emphasize that for this to be successful, in addition to further education, there needs to be sensible allocation of resources. Ideally, all interventions would be led by a specialist team, but due to limited resources this is not likely to be possible everywhere. A universal management algorithm, with supplementary information for patients and their carers, would allow a more generalist team to manage it adequately. Even with this in place, the complexity of the patient cohort, with contributing issues such as cachexia, sarcopenia, and delirium, will continue to cause potentially unavoidable challenges in some instances.

Although most participants in this survey believe they have adequate time to successfully manage fatigue, to achieve a sustained improvement more time may be needed than anticipated. Results justify the need for regular education of staff, patients, and carers; an individualized documented management plan for each patient that will need to be reviewed as the patient's condition changes; regular monitoring to assist with motivation and compliance; and regular access to allied health services. Given that most interventions need to be initiated and maintained in the community setting, this also needs to be considered. Further studies would need to ensure that any guideline could be replicated in the community setting, for both cancer and noncancer patients. Although fatigue-specific services and individualized management plans are yet to be trialed for fatigue management, they are frequently and effectively used in the management of dyspnea and should be considered a viable option to address this need.^14^

There is only weak evidence available in the literature (largely level II/III data) to guide current management^5^ and the majority targets cancer patients on treatment or cancer survivors, not all of whom are PC patients. There is some literature available for the HIV, multiple sclerosis, and chronic obstructive pulmonary disease (COPD) populations,^15^ but on the whole, other noncancer conditions that are increasingly managed in the PC setting are not represented. It is recognized that fatigue is still a major issue in these populations^1^ and it is assumed that available evidence is transferrable to these groups.

National guidelines, such as the NCCN and National Institute for Health and Care Excellence (NICE), show inconsistent recommendations, but the general consensus is that nonpharmacological strategies should be prioritized; pharmacological interventions are not recommended except in selected individuals.^5^ A Cochrane systematic review by Mucke et al. included the review of 45 studies (both malignant and nonmalignant diagnoses) and concluded that there was insufficient evidence to support the use of specific medications to treat fatigue in PC patients, although there was slightly superior effect of methylphenidate compared with placebo in CRF.^6^

Currently, exercise and CBT are the two interventions with the best supporting evidence. This study suggests that within the SLHD, exercise is being suggested, but what exercises specifically remains unclear. CBT is being used infrequently, possibly due to a lack of awareness of its benefit. Guidance suggests that exercise programs must be individualized to the patient and include a mixture of strength/resistance and aerobic exercises. Despite most studies incorporating intensive programs, compliance and patient satisfaction appear to be high. Cheville et al. randomized 66 patients with stage IV lung or colorectal cancer to either usual care or incremental walking and home-based strength training. The protocol suggested exercising four or more times a week, with an initial physiotherapy visit and bimonthly follow-up phone calls. At week eight, the primary endpoint mobility (*p* = 0.01) and secondary endpoints, fatigue (*p* = 0.02) and sleep quality (*p* = 0.05), all improved compared with usual care.^16^ Whether the level of support offered in this study can be replicated sustainably for community PC patients remains uncertain, and if not, the question is whether such positive results could be replicated. Participants' performance status was also likely better than many PC patients.

The evidence behind CBT largely comes from the cancer survivor population^17^; these data have been extrapolated to the PC setting, but as yet there are no randomized-controlled trials.^18^ The assumption is that fatigue-related cognitions such as low self-efficacy and catastrophizing thoughts or behaviors such as poor sleep hygiene lead to the persistence of fatigue. If CBT successfully reformulates dysfunctional thoughts or behaviors, there will be a reduction in the severity of and/or distress from fatigue.^18^

### Limitations of the study

The main limitations are as expected with a study of this size and type; this includes small sample size, limited study population, and incomplete responses. Because this is a survey-based study (not semistructured interviews), certain themes may have been missed and/or not explored in sufficient detail. We also did not establish whether the nurses were generalist or specialist PC nurses. There had been increased discussion and education about fatigue management predata collection, which may have contributed to bias.

### Areas for further research

The outcomes of this survey support the need for further research to establish the most effective way to improve fatigue management for PC patients, including intervention type and delivery. Consideration needs to be given to what would be possible given the expected resource allocations, and whether a multimodal community-based intervention is appropriate. If so, this needs to be incorporated more systematically in future guidelines. There also needs to be a focus on improving HCP understanding and knowledge, and how this could be best achieved.

## Conclusion

This survey-based study confirms that HCPs lack confidence in assessing and completing an individualized management plan for patients with a life-limiting illness and distressing levels of fatigue. With no universal guideline, the approaches adopted are highly variable. It justifies the need for further education for all HCPs involved in the care of palliative patients, and further research aimed at assessing the effectiveness of a multimodal intervention within the confines of finite resources in the community setting.

## Abbreviations Used

CALD: culturally and linguistically diverse
CBT: cognitive-behavioral therapy
CRF: cancer-related fatigue
EAPC: European Association of Palliative Care
HCPs: health care professionals
NCCN: National Comprehensive Cancer Network
NICE: National Institute for Health and Care Excellence
OT: occupational therapy
PC: palliative care
PCOC: Palliative Care Outcomes Collaborative
PCU: palliative care unit
SAS: symptom assessment scale
SLHD: Sydney Local Health District

## References

[1] Radbruch L, Strasser F, Elsner F, et al.: Fatigue in Palliative Care Patients—An EAPC approach. Palliat Med 2008;22:13–32 18216074 10.1177/0269216307085183

[2] Connolly A, Allingham S, Bishop G, et al.: *Patient Outcomes in Palliative Care in Australia, National Compendium Report, July to December 2016*. PCOC, Australian Health Services Research Institute, University of Wollongong, 2017

[3] Stone P, Richardson A, Ream E, et al.: Cancer related fatigue: Inevitable, unimportant and untreatable? Results of a multi-centre patient survey. Ann Oncol 2000;11:971–975 11038033 10.1023/a:1008318932641

[4] Vogelzang NJ, Breitbart W, Cella D, et al.: Patient, caregiver and oncologist perceptions of cancer related fatigue: Results of a tripart assessment survey. Semin hematol 1997;34:4–12 9253778

[5] Vilchynska T, Beard B: Cancer-related fatigue in palliative care: A global perspective. Int J Palliat Nurs 2016;22:244–252 27233012 10.12968/ijpn.2016.22.5.244

[6] Mucke M, Mochamat, Cuhls H, et al.: Pharmacological treatments for fatigue associated with palliative care: Executive summary of a Cochrane Collaboration systematic review. JCSM 2016;7:23–27 10.1002/jcsm.12101 PMC4799864 27066315

[7] A Profile of Patients Receiving Palliative Care, Sydney District Nursing report, January to June 2018. PCOC, Australian Health Services Research Institute, University of Wollongong, 2018

[8] Pearson EJM, Morris ME, McKinstry CE: Cancer-related fatigue: A survey of health practitioner knowledge and practice. Support Care Cancer 2015;23:3521–3529 25847296 10.1007/s00520-015-2723-8

[9] Etikan I, Musa SA, Alkassim RS: Comparison of convenience sampling and purposive sampling. Am J Theor Appl Stat 2016;5:1–4

[10] Pope C, Ziebland S, Mays N: Qualitative research in health care. Analysing qualitative data. BMJ 2000;320:114–116 10625273 10.1136/bmj.320.7227.114 PMC1117368

[11] Williams LA, Bohac C, Hunter S, et al.: Patient and health care provider perceptions of cancer-related fatigue and pain. Support Care Cancer 2016;24:4357–4363 27207616 10.1007/s00520-016-3275-2 PMC4993798

[12] Passik SD, Kirsh SL, Donaghy K, et al.: Patient-related barriers to fatigue communication. J Pain Symptom Manage 2002;24:481–493 12547048 10.1016/s0885-3924(02)00518-3

[13] Cancer-Related Fatigue: National Comprehensive Cancer Network Clinical Practice guidelines in Oncology. 2017, version 2 10.6004/jnccn.2003.0029 19761067

[14] Bausewein C, Schunk M, Schumacher P, et al.: Breathlessness services as a new model of support for patients with respiratory disease. Chronic Respir Dis 2018;15:48–59 10.1177/1479972317721557 PMC5802660 28718321

[15] Payne C, Wiffen PJ, Martin S: Interventions for fatigue and weight loss in adults with advanced progressive illness. Cochrane Database Syst Rev 2012;1:CD008427 22258985 10.1002/14651858.CD008427.pub2

[16] Cheville AL, Kollasch J, Vandenberg J, et al.: A home-based exercise program to improve function, fatigue and sleep quality in patients with stage IV lung and colorectal cancer: A randomised controlled trial. J. Pain Symptom Manag 2013;45:811–821 10.1016/j.jpainsymman.2012.05.006 PMC4524515 23017624

[17] Jacobsen PB, Donovan KA, Vadaparampil ST, et al.: Systematic review and meta-analysis of psychological and activity based interventions for cancer related fatigue. Health Psychol 2007;26:660–667 18020836 10.1037/0278-6133.26.6.660 PMC2398706

[18] Poort H, Verhagen CA, Peters ME, et al.: Study protocol of the TIRED study: A randomised controlled trial comparing either graded exercise therapy for severe fatigue or cognitive behaviour therapy with usual care in patients with incurable cancer. BMC Cancer 2017;17:81 28129746 10.1186/s12885-017-3076-0 PMC5273841

